# Detachment of ligands from nanoparticle surface under flow and endothelial cell contact: Assessment using microfluidic devices

**DOI:** 10.1002/btm2.10089

**Published:** 2018-04-17

**Authors:** Maria Jarvis, Michael Arnold, Jenna Ott, Vinu Krishnan, Kapil Pant, Balabhaskar Prabhakarpandian, Samir Mitragotri

**Affiliations:** ^1^ Biomolecular Sciences and Engineering Program University of California Santa Barbara CA 93106; ^2^ Dept. of Molecular, Cellular and Developmental Biology University of California Santa Barbara CA 93106; ^3^ Dept. of Chemical Engineering University of California Santa Barbara CA 93106; ^4^ Biomedical Technology, CFD Research Corporation Huntsville AL 35806; ^5^ John A. Paulson School of Engineering and Applied Sciences Wyss Institute, Harvard University Cambridge MA 02138

**Keywords:** degradation, ligands, microfluidic device, microvascular, nanoparticles, shear, targeting, translation

## Abstract

Surface modification of nanoparticles is a well‐established methodology to alter their properties to enhance circulation half‐life. While literature studies using conventional, in vitro characterization are routinely used to evaluate the biocompatibility of such modifications, relatively little attention has been paid to assess the stability of such surface modifications in physiologically relevant conditions. Here, microfluidic devices were used to study the effect of factors that adversely impact surface modifications including vascular flow and endothelial cell interactions. Camptothecin nanoparticles coated with polyethylene glycol (PEG) and/or folic acid were analyzed using linear channels and microvascular networks. Detachment of PEG was observed in cell‐free conditions and was attributed to interplay between the flow and method of PEG attachment. The flow and cells also impacted the surface charge of nanoparticles. Presence of endothelial cells further increased PEG shedding. The results demonstrate that endothelial cell contact, and vascular flow parameters modify surface ligands on nanoparticle surfaces.

## 1 INTRODUCTION

Numerous nanoparticles have been developed over the years for therapeutic applications where they hold great promise for effective therapeutic drug delivery, primarily in oncology.^1^, ^2^ These nanoparticles include polymeric systems, liposomes, micelles and nanocrystals, among others.^3^, ^4^, ^5^, ^6^ Regardless of the composition of nanoparticles, their surface is often modified to extend their blood circulation using polyethyleneglycol (PEG) for instance, or with targeting ligands to enhance tissue‐targeting.^7^, ^8^, ^9^, ^10^ A large number of studies have demonstrated the ability of PEG to minimize opsonization and clearance by the reticulo‐endothelial system sequestration.^9^, ^11^, ^12^ At the same time, several targeting ligands including small molecules, peptides, and antibodies have been successfully used for targeting purposes.^11^, ^12^, ^13^, ^14^

A number of studies have focused on the design and characterization of surface coating of nanoparticles.^15^, ^16^ For example, studies have focused on characterization and optimization of PEG coating and surface ligand density.^17^, ^18^, ^19^ These surface coatings are often developed based on in vitro studies and relatively little is known about their stability in vivo. In addition, close contact of these nanoparticles with endothelial cells in vivo may also adversely impact their stability. In this study, we systematically assess the role of vascular flow and endothelial cells in the stability of surface coating on nanoparticles using endothelial cell‐laden microfluidic devices.

The nanoparticles used in this study were rod‐shaped camptothecin (CPT) nanocrystals,^20^, ^21^ modified with PEG which was physically or chemically tethered to the surface. Previous studies in our and other laboratories have demonstrated the utility of nanocrystals for therapeutic applications.^22^, ^23^, ^24^, ^25^, ^26^, ^27^ An additional variant of CPT nanocrystal, carrying PEG‐folic acid (FA) conjugate was also investigated. These nanocrystalline camptothecin‐based nanoparticles were selected as a test particle group primarily based on the previous research performed on these particles in our laboratory. Similar studies could be performed using other nanoparticle types. Two types of microfluidic devices were used; microvascular network devices (MNs) that mimic complex vasculature and fluid flow conditions observed in vivo or linear channel device's (LCs) which offer a simple and constant flow system.

## 2 METHODS

### 2.1 Preparation and analysis of camptothecin nanocrystals

**Figure 1:**
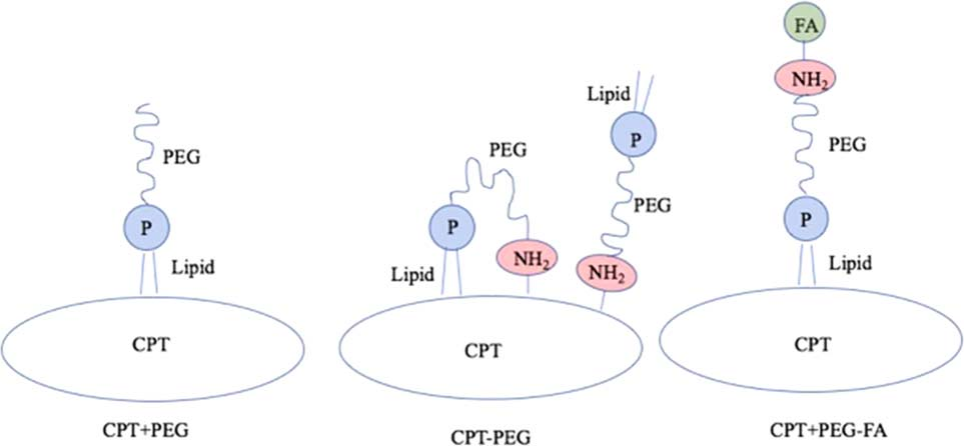
Schematic representation of three CPT nanoparticle scaffolds prepared and used in this study. In case of CPT + PEG, the lipid chain of DSPE‐PEG is expected to noncovalently associate with the hydrophobic surface of CPT as shown in the schematic. In case of CPT‐PEG, two configurations are likely; the amine group in DSPE‐PEG‐amine is chemically conjugated to the surface of CPT, thus exposing the lipid chain outside. DSPE‐PEG‐amine may also fold due to hydrophobic interactions between the lipid chain and hydrophobic CPT surface leading to anchoring of the lipid chain on the CPT surface thus exposing PEG in a loop. For CPT + PEG‐FA, the DSPE‐PEG‐FA is expected to anchor on CPT by its hydrophobic tail, thus exposing FA outwards

All CPT nanocrystals were prepared using the solvent diffusion method as described previously^20^ as shown in Figure 1.

#### 2.1.1 CPT + PEG nanoparticles (PEG physically adsorbed on CPT)

About 5 ml of 0.8 mg/ml of CPT (Sigma Aldrich) and 3.2 mg/ml DSPE PEG2K Amine (Avanti Polar Lipids) in DMSO solution were pipetted dropwise into a 120 ml water mixture containing 1% w/w alpha‐tocopherol (Sigma). The mixture was stirred at 800 rpm under constant ultrasonication at room temperature (22°C) for 1 hr. CPT + PEG nanocrystals formed at the boundary where DMSO diffused into the water. The CPT + PEG nanocrystals were then centrifuged three times at 20°C with milliQ water (18.2 MΩ•cm) at 3,500 rpm. The concentration of Camptothecin in CPT + PEG nanocrystals was determined by dissolving the nanocrystals in DMSO and reading the absorbance at 366 nm using a spectrophotometer (Tecan M220 Infinite Pro). The successful incorporation of DSPE PEG2K Amine was validated via X‐Ray diffraction of DSPE PEG2K Amine, CPT, and alpha‐tocopherol in their free powder form compared to the CPT + PEG construct (Panalytical Empyrean Powder Diffractometer). DSPE PEG2K Amine content was analyzed using an inductively coupled plasma (ICP) Atomic Emission Spectrometer (Thermo iCAP 6300 Model) the detection of Phosphorous signals using calibration curves prepared with a Phosphorous Standard for ICP (TraceCERT, 1,000 mg/L P in H_2_O, Sigma).

#### 2.1.2 CPT + PEG‐FA (PEG‐FA physically adsorbed on CPT)

DSPE PEG2K Amine‐FA conjugates were prepared first. Specifically, 4.5 mg of FA was dissolved in 500 µl of DMSO. This solution was then added to 100 µl of 5 mg/ml EDC (Sigma) in DMSO solution. It was then vortexed and stirred for 30 min at room temperature. To this solution, 19 mg of DSPE PEG2K Amine dissolved in 500 µl of DMSO was added; this combined mixture was vortexed and rotated overnight at room temperature. The DSPE PEG2K Amine–Folic Acid conjugate was then purified with a HyperSep C18 octadecyl uncapped bonded silica column, with an acetonitrile‐milliQ H_2_O (18.2 MΩ•cm) 5–50% v/v gradient. Polymer–Folic acid conjugate eluents were then analyzed via Matrix Assisted Laser Desorption Ionization—Mass Spectrometery (MALDI—MS, Microflex LRF A Bruker) and with FTIR set to 24 scans and taken in acetonitrile (Magna IR 850 Nicolet). FTIR spectra were analyzed in the fingerprint region using OMNIC software.

To incorporate PEG‐FA into CPT nanoparticles, 5 ml of 0.8 mg/ml of CPT (Sigma Aldrich) in DMSO solution was pipetted dropwise into a 120 ml water mixture containing 1% w/w alpha‐tocopherol (Sigma). About 20% Acetonitrile‐milliQ H_2_O (18.2 MΩ•cm) eluent containing PEG‐FA conjugate was then added dropwise to this solution and the overall mixture containing CPT, DSPE PEG2K Amine, and FA was stirred at 800 rpm with constant ultra‐sonification at room temperature (22°C) for 1 hr. After 1 hr, the CPT + PEG‐FA nanocrystals were then centrifuged three times at 20°C with milliQ water (18.2 MΩ•cm) at 3,500 rpm. Presence of folic acid was quantified using absorbance at 290 and 370 nm, and CPT was quantified using fluorescence at 366/434 nm—both utilized a spectrophotometer (Tecan M220 Infinite Pro). DSPE PEG2K Amine was quantified using ICP‐MS as used for CPT + PEG constructs.

#### 2.1.3 CPT‐PEG nanoparticles (PEG chemically conjugated to CPT)

To prepare CPT‐PEG, unmodified Camptothecin nanocrystal surfaces were activated with carbonyldiimidazole (CDI, MR 1:10, Sigma) in pH 7.4 1x PBS buffer for 5 min. The activated particles were then centrifuged at 5,000 rpm, for 30 min at 20°C and washed three times using DI water. They were then mixed with DSPE PEG2K Amine (PEG‐NH_2_, MR 1:3) in 1x PBS pH 7.4 and left for overnight coupling at 4°C. Finally, the unreacted PEG‐amine was removed after three washes with DI water.

In some experiments, FA was directly conjugated to CPT (CPT‐FA). To prepare these particles, 600 µl of 0.5 mg/ml of unmodified CPT nanocrystals were added to 400 µl of the CDI stock solution. This solution was allowed to rotate for 15 min at room temperature. CDI‐activated CPT nanocrystals were spun down at 5,000 rpm for 15 min at room temperature. Pellets were collected, washed with DI water, and resuspended in a 0.8 mg/ml solution of Folic Acid in MQH_2_O at pH 5. Folic acid and CDI activated CPT nanocrystals were incubated together and rotated overnight at 4°C. CPT‐FA were then spun down at 5,000 rpm, 30 min at 20°C and washed two times at these conditions in MQH_2_O. CPT and FA presence were determined via absorbance at 366 and 290 nm respectively using independent CPT and FA standard curves on the Tecan M200 PlateReader.

Morphologies of CPT + PEG, CPT‐PEG, and CPT + PEG‐FA nanocrystals were analyzed using a scanning electron microscope (SEM). Surface charges of all nanocrystalline scaffolds suspended in 1x PBS pH 7.4 were measured as zeta potential (ZP) using a Nanoseries‐Zetasizer (Malvern).

### 2.2 Cell culture

Human endothelial cell line, EA.hy926, commercially obtained from ATCC were cultured in DMEM medium supplemented with 10% FBS and 1% Penicillin–Streptomycin (Pen–Strep) in a humidified incubator with 5% CO_2_ at 37°C. Confluent cells were subcultured in 1:3 ratio and used for all experiments.

### 2.3 Microfluidic device preparation for nanocrystalline physical studies

**Figure 2:**
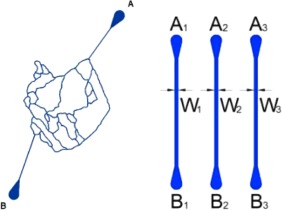
Schematic depictions of the microvascular networks (MNs; 100 × 100 μm^2^) and the linear channel devices (LCs; 100 × 250 μm^2^). Channels represented in blue

MNs and LCs devices were purchased from SynVivo (Cat #105002,101002, Figure 2). The MNs have a constant width and depth of 100 µm while the LCs have a constant width of 250 µm and depth of 100 µm. MNs and LCs were coated with 100 µg/ml human fibronectin (Thermo Fisher), subjected to 5 PSI N_2_ (laboratory grade) for 15 min, and incubated in a humidified incubator with 5% CO_2_ at 37°C in preparation for seeding with EA.hy926 cells.

Once seeded, EA.hy926 cells were allowed to incubate for 4 hr to attach to the channels of the MNs before changing the media using a syringe pump (KD Scientific Inc.). For the duration of the experiments (*n* = 5 days), Eahy.926 cells received media changes every 12 hr at 2 µl/min for 10 min using a syringe pump (KD Scientific Inc.). Freshly prepared CPT PEG, CPT‐PEG, or CPT PEG‐FA nanocrystals were then infused through the vascular channels at 0.5 mg/ml, 4 µl/min, for 2 hr in MNs (max shear stress: 3.7 dyne/cm^2^, min shear stress: 1.86 dyne/cm^2^) and LCs (shear stress: 1.1 dyne/cm^2^). Shear stresses and ranges calculated using the dynamic viscosity of DMEM (0.00089 Pa s) and methods developed by Smith et al.^28^, ^29^ Nanocrystals were collected and saved for further analysis. MNs were washed with 1× PBS at 2 µl/min for 15 min and imaged post infusion using an inverted microscope (Olympus CKX‐41). For control studies performed in MNs and LCs lacking endothelial cells, the devices were subjected to the same preparative conditions as described above.

### 2.4 Endothelial cell viability within MNs and LCs

Cell viability prior to the infusion of nanocrystals within the MNs was analyzed using SYTOX Green Nucleic Acid Stain and LYSOTracker Red DND (Invitrogen). EA.hy926 cells were seeded and cultured in MNs at a density of 100,000 cells/ml in DMEM, 10% FBS, 1% Pen–Strep. Cells were allowed to attach overnight and grown for *n* = 3 days before administering cell viability dyes at 4 µl/min for 30 min. Fluorescent images were taken using FITC and TRITC filters on an Olympus CKX‐41 followed by processing and quantitation using ImageJ.

MNs and LCs were also imaged using phase contrast on brightfield settings on day 5 post seeding and after unmodified CPT nanocrystals infusion at 4 µl/min for 500 µl. Post infusion device channels were washed with DMEM, 10% FBS, 1% Pen–Strep at 2 µl/min for 50 µl. Devices were tested for the cell concentration within their channels as a means for measuring their survivability and ability to withstand the flow forces they were conditioned under. Cells were imaged under 10× and 4× on an Olympus CKX‐41 and a Zeiss Axiovert 25. Images were obtained as qualitative evidence for endothelial cell presence pre‐ and post‐flow within the LCs and the MNs, respectively. All images were quantified using the Cell Counter plug in available on ImageJ, *n* = 4 regions of interest were selected for two‐way ANOVA statistical analyses performed on GraphPad Prism 7.

### 2.5 Analysis of CPT nanocrystals pre‐ and post‐infusion

CPT + PEG, CPT‐PEG, and CPT + PEG‐FA nanocrystals were analyzed pre‐ and post‐infusion through MNs and LCs seeded with and without EA.926 cells. Pre‐infusion and post‐infusion analyses utilized ZP measurements (Malvern Zetasizer Nano ZS) and Phosphorous detection (Thermo iCAP 6300 ICP) for PEG coating, content, and shearing analyses. Differences in ZP and phosphorous content were analyzed using paired *t* tests available in GraphPad Prism 7, degrees of freedom and P values reported in Results.

## 3 RESULTS

**Figure 3:**
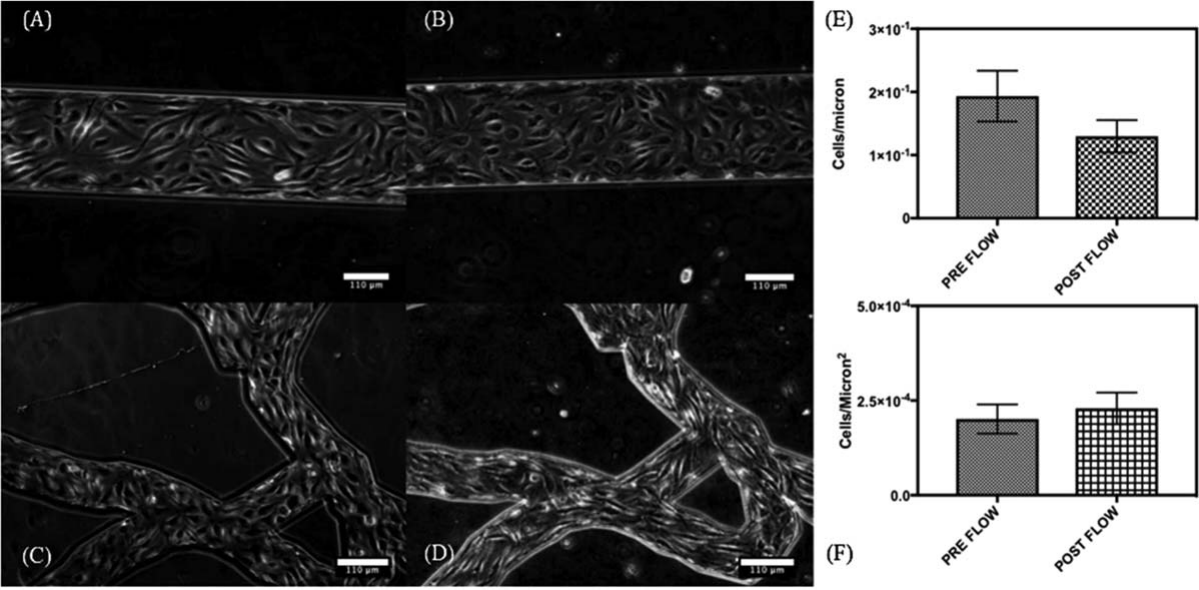
Brightfield images of a LC and MN device seeded with EAhy926 taken 5 days post initial seeding (Pre‐Flow) and after nanocrystal infusion (Post‐flow). Images were processed using ImageJ. (A) LC pre‐flow (B) LC post flow (C) MN pre‐flow, (D) MN post flow images taken at 10×. Cell numbers quantified for (E) LCs and (F) MNs, standard deviation represented on graphs

All CPT nanocrystals were flown through MNs and LCs at 4 μl/min. These flow rates were selected to maintain similar flow conditions and shear stresses within the devices that were sufficiently low so as to not cause endothelial cell detachment while also maintaining the lower range limit of physiologically relevant shear stresses associated with diseased vasculature.^20^, ^30^, ^31^ Due to complex bifurcations, loops, and intersections, the shear stresses at the core of the MNs are expected to drop with a return to inlet level shear stresses upon the reunification of the bifurcated channels into the linear portion leading to the exit port.^32^ Microvascular network devices were seeded with endothelial cells and conditioned under constant flow for 5 days before cell viability was tested using live/dead fluorescent probes. Post infusion, MN, and LC devices were imaged under brightfield phase contrast to verify the presence of endothelial cells at pre‐flow confluency counts (Figure 3).

CPT nanocrystals were collected after flow through the devices and were centrifuged to form a pellet. Phosphorous content (arising from DSPE‐PEG) in the supernatant and the pellet was separately determined using ICP spectrometer. Detachment of PEG from nanoparticle surface was assessed by quantifying the ratio of phosphorous in the supernatant and the pellet. Freshly prepared CPT (CPT + PEG, CPT‐PEG, and CPT + PEG‐FA) showed no detectable amounts of phosphorous in the supernatant.

**Figure 4:**
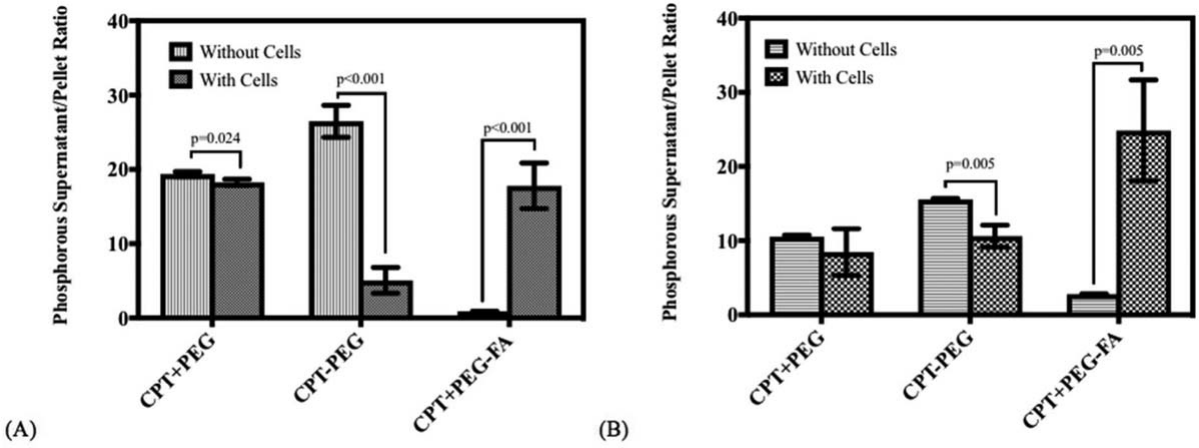
Supernatant to pellet ratios of phosphorous concentration (measured individually in ppm or g/L) post flow in (A) LCs with and without endothelial cells, EA.hy926 and (B) in MNs with and without endothelial cells, paired *t*‐test, *n* = 5

Flow as well as presence of endothelial cells induced significant detachment of PEG from the nanocrystals (Figure 4A). Physically adsorbed PEG (CPT + PEG) was readily desorbed from the surface as indicated by high supernatant‐to‐pellet ratio (Figure 4A). This number was nearly the same with and without the cells, indicating that desorption of PEG from CPT + PEG was mediated by the device or flow, and not endothelial cell‐contact.

Interestingly, removal of PEG was also observed in the case of chemically attached PEG (CPT‐PEG, Figure 4A). In the absence of cells, majority of PEG was removed from CPT‐PEG. Note however, that the detachment was dramatically reduced in the presence of cells (Figure 4A, CPT‐PEG dark gray bar). Strikingly different behavior was observed for CPT + PEG‐FA. Specifically, flow through blank LCs induced little detachment of PEG (Figure 4A). The level of detachment was strikingly increased when CPT + PEG‐FA nanocrystals were flown through endothelial cell‐laden devices.

The effect of flow and endothelial cell contact was qualitatively similar and followed the same general trends for MNs and LCs (Figure 4A,B), although the extent of PEG detachment in MNs was generally lower compared to that observed for LCs, with the exception of CPT + PEG‐FA in endothelial‐cell laden MNs. The results suggest that the ligand detachment is a combined result of flow and cellular contact.

**Figure 5:**
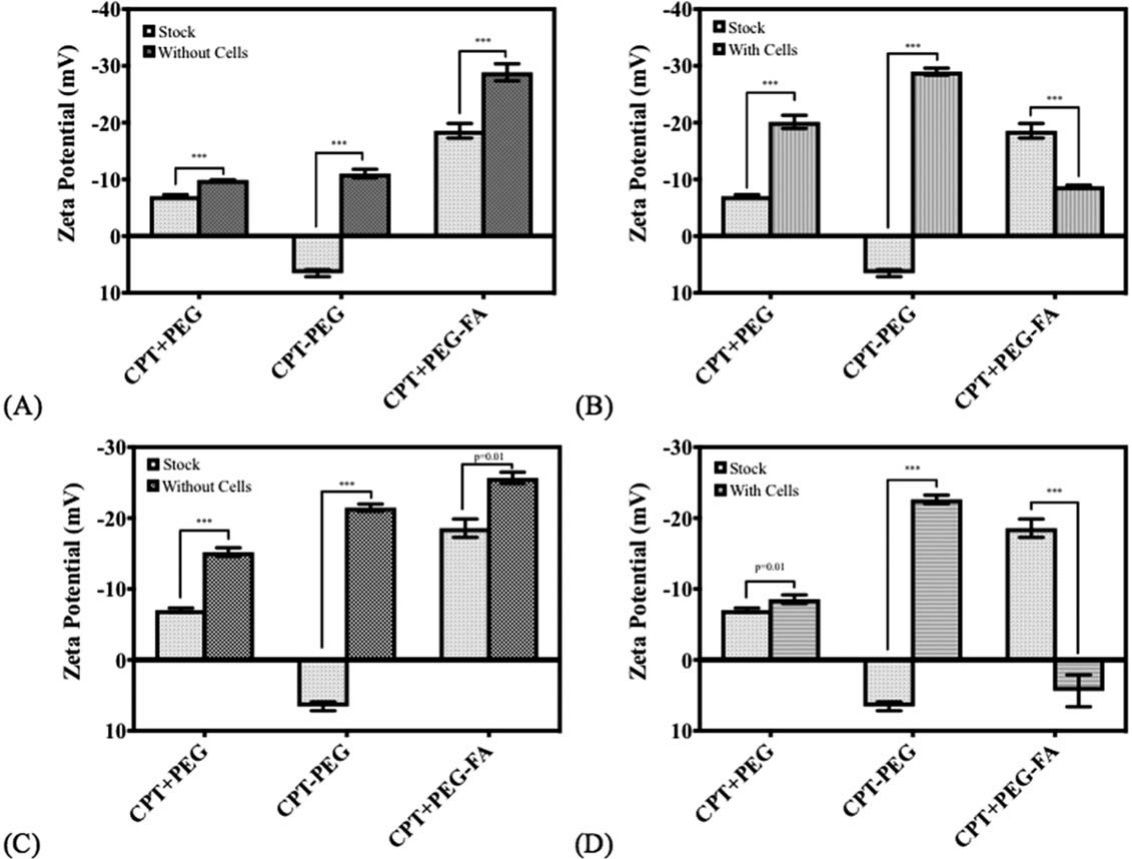
Changes in ZP on the surface of the nanocrystalline pellet measured post flow for (A) LCs with a base fibronection coating, (B) LCs seeded with endothelial cells, (C) MNs with a base fibronectin coating, and (D) MNs seeded with endothelial cells. Paired *t*‐test, *n* = 3 for all conditions. ***p < .01

Surface charge of nanoparticles also exhibited significant change upon flow through the devices. In the absence of endothelial cells, relatively small, but statistically significant decrease in ZP of CPT + PEG was observed (Figure 5A). Specifically, the ZP of CPT + PEG particles decreased from −9.89 ± 0.02 mV to −7.03 ± 0.28 mV after flow through LCs. Significantly greater change of similar nature was observed for CPT‐PEG nanocrystals. Specifically, the ZP of stock CPT‐PEG nanocrystals was strongly positive (+6.55 ± 0.62 mV) which decreased significantly (−11.03 ± 0.75 mV) after flow through LCs. A significant change was also observed for CPT + PEG‐FA (increase from −18.57 ± 1.29 mV to −28.87 ± 1.50 mV). Inclusion of endothelial cells generally amplified these trends, except for CPT + PEG‐FA where the flow through devices led to significant increase in ZP, suggesting either significant cell‐mediated removal of FA from the PEG‐FA conjugates or mass agglomeration of folic acid head groups from CPT + PEG‐FA. The trends observed in MNs were qualitatively and quantitatively similar to those observed for LCs. (Figure 5B). The changes observed in MNs were qualitatively similar to those observed in LCs (Figure 5C,D). Taken together these matching trends support the core significant findings of this work which depict the reversal of detachment patterns upon the addition of a ligand which is chemically visible to the endothelial cell surface.

## 4 DISCUSSION

This study used a combination of microfluidic devices to probe the effects of flow and endothelial cell contact on ligand shedding from nanoparticles. Camptothecin nanocrystals were used as a test particle system for use within the microfluidic devices. Several studies report the use of microfluidic devices for studying tumor microenvironments, target‐screening, and cancer metastases modeling.^33^, ^34^, ^35^, ^36^, ^37^, ^38^, ^39^, ^40^ This study aims to use microfluidic devices to specifically probe the combined effects of complex architecture and flow‐induced shear/wall contact by nanocrystals and other drug delivery carriers during flow in the vascular network. PEG was attached to camptothecin nanocrystals either via chemical conjugation (CPT‐PEG) or physical adsorption (CPT + PEG). The third scaffold involved the conjugation of a PEG to folic acid (PEG‐FA), which has been previously shown to target breast cancer cells.^41^ The PEG‐FA conjugates were physically adsorbed to the surface of a camptothecin nanocrystal (CPT + PEG‐FA).

Two types of microfluidic devices were used in this study, LCs which simply provide a means of imposing flow on the nanocrystals and MNs which provide the complex connectivity of channels routinely observed within in vivo MNs. (Figure 2). Both devices were able to grow cells and maintain viability under flow for extensive time periods (Supporting Information Figures 2 and 3, Figure 3). Flow of nanocrystals itself did not adversely impact the endothelial cells, thus confirming the suitability of the devices for screening effect on particles. All pegylated nanocrystals were flown through microfluidic devices seeded with or without endothelial cells. Detachment of PEG from nanocrystals was assessed by measuring phosphorous using ICP‐AS taking advantage of the fact that each strand of PEG contained one phosphorous atom.

Significant detachment of PEG was observed from nanocrystals in response to flow and endothelial cell‐contact (Supporting Information Figure 3, Figure 4). The extent of removal depends on several factors including the nature of attachment of PEG to the surface, presence of cells, presence of a targeting ligand and presentation of PEG. Specifically, physically adsorbed PEG exhibited extensive shedding due to flow and the extent of shedding was not significantly impacted by the presence of cells. This is consistent with the fact that PEG is unlikely to exhibit significant interactions with endothelial cells, thus leaving flow‐induced wall contact or flow‐induced shear as the primary source of PEG detachment. Peculiar observations were made for chemically conjugated PEG (CPT‐PEG); in the absence of cells, chemically conjugated PEG exhibited higher shedding than physically adsorbed PEG. This observation is counter‐intuitive, although it may have originated from the differences in the orientation of PEG on the nanocrystal surface (Figure 1). Chemically conjugated PEG may potentially extend its lipid chain under flow and induce detachment due to the interactions of the lipid tail with the wall which is consistent with findings describing tethered polymer extensions subjected to flow, a phenomena witnessed under flow described as critical stretching.^42^, ^43^ In the presence of cells, PEG shedding was significantly reduced possibly due to reduced interactions of PEG with the cells. The most striking differences were found for PEG‐FA‐coated nanoparticles. In the absence of cells, CPT + PEG‐FA was the most resilient coating, exhibiting little shedding. In the presence of cells, this was significantly reversed, leading to extensive shedding, likely due to direct interactions of FA with endothelial cells.

All particles exhibited a significant change in ZP due to passage through the device (Figure 5A,C). CPT + PEG (physical attachment) and CPT‐PEG (chemical attachment) exhibited a decrease in ZP post‐flow whereas CPT + PEG‐FA particles exhibited an increase in the potential (Figure 5B,D). Negative ZP shifts post‐flow likely arises from the loss of surface PEG, thus exposing the unmodified camptothecin nanocrystalline surface which has a negative ZP.^20^ Positive ZP shifts post‐flow possibly arose due to exposure of the amine group at the end of the PEG which was previously engaged in the amide bond to the carboxyl group of the folic acid. It is possible that with the exposure of the positively charged amine group, there was an increase in head group aggregation which has been observed previously in microvascular network device studies probing cationic nanopolymers resulting in significant overall surface charge increases.^44^

The results presented here clearly show that flow and flow‐induced contact with the wall (either bare wall or and endothelial cells) offer sources of ligand shedding from nanoparticle surface. At the same time, the same factors can also induce significant changes in nanoparticle structure. The final extent of ligand shedding is likely determined by the balance of its interactions with the nanoparticle surface and vascular surface whereas the flow is likely to be an accelerator or a mediator of ligand re‐distribution. Physically adsorbed ligands in principle are more susceptible to ligand shedding, however, even chemically conjugated ligands are susceptible to shedding if the interactions with the wall/endothelial cells are dominant. Care should be taken while interpreting the extent of ligand removal observed in this study. Some of the ligand on the particle may actually potentially be simply loosely adsorbed. While we removed unbound ligand by centrifugation after synthesis, it is possible that some ligands are physiosorbed on the nanoparticle, do not come off during centrifugation, but come off with the flow. Further, note that the ligands are attached to the nanoparticle surface which itself is susceptible to dissolution. Hence, it is possible that flow loosen the attached ligands which are then completely removed during centrifugation post‐flow. In other words, centrifugation may enhance the measured effect of flow in the device. In view of this possibility, we suggest that emphasis should be placed on the trends rather than the actual fraction deemed desorbed from the analysis. It is also possible that the shedding of the ligand in the devices is determined by some specific critical locations of high shear or cellular contact, rather than average uniform shear throughout the device. The extent of ligand‐shedding will depend on several nanoparticle parameters including material, size, shape, and deformability. Fragility of the particles may also play a role. Conjugation chemistry, especially the length of the linker and the strength of the covalent bond will also impact the degree of shedding. Finally, the chemistry of the ligand, especially the strength of interaction with the vascular wall as well as that with the nanoparticle surface will also impact the extent of detachment. Specifically, flow of the particles in the devices exposes to the high surface area of the vascular wall, which depending on its chemistry, may induce redistribution of the ligand depending on the chemistry. Studies should be performed in future to fully understand the extent of ligand detachment. While the extrapolation of results presented here to other nanoparticles should be done with caution, the findings clearly demonstrate the necessity of assessing these issues during translation of nanoparticles from in vitro to in vivo studies.

## Supporting information

Additional Supporting Information may be found online in the supporting information tab for this article.

Supporting Information
