# Supplementary material for: Detachment of ligands from nanoparticle surface under flow and endothelial cell contact: Assessment using microfluidic devices
Source: Bioeng Transl Med. 2018 Apr 17;3(2):148–55. doi: 10.1002/btm2.10089 (PMC6063868; doi:10.1002/btm2.10089)
Supplement: Supplementary file 1 — Supporting Information [file BTM2-3-148-s001.docx]

**
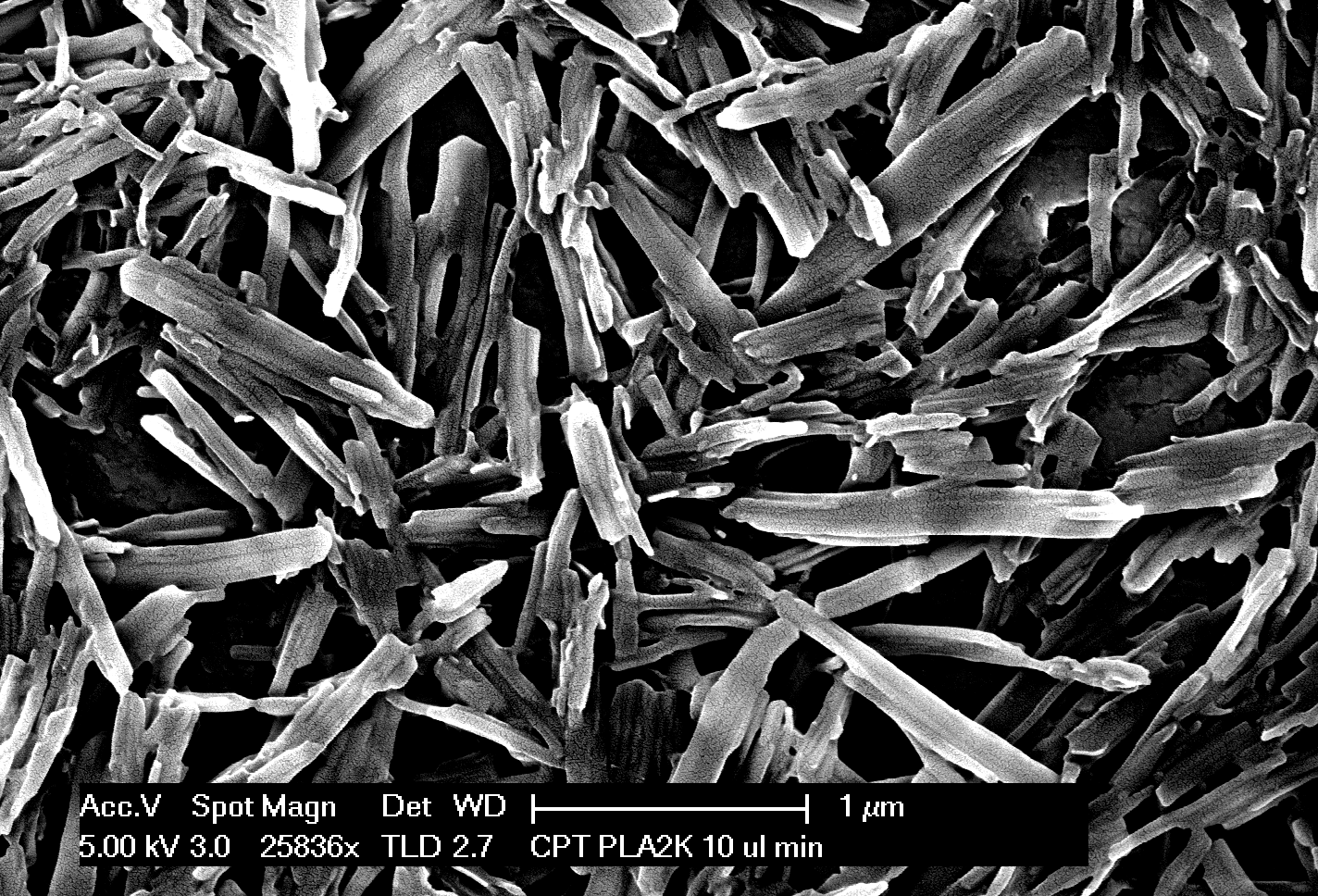

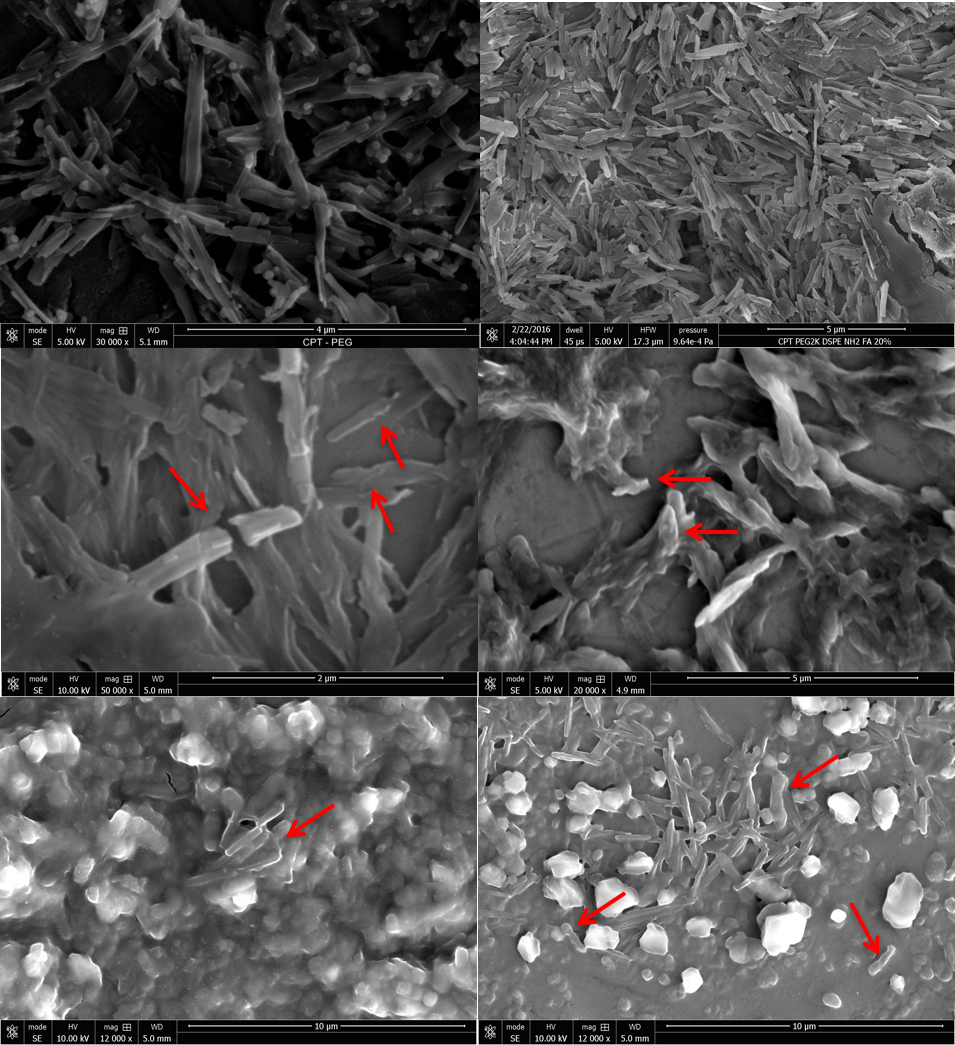
**

**Figure S1. Figure 1. Left to Right, Scanning electron microscopy (SEM) showing the morphology and size of CPT+PEG, CPT-PEG, and CPT+PEG -FA.**

**(a)
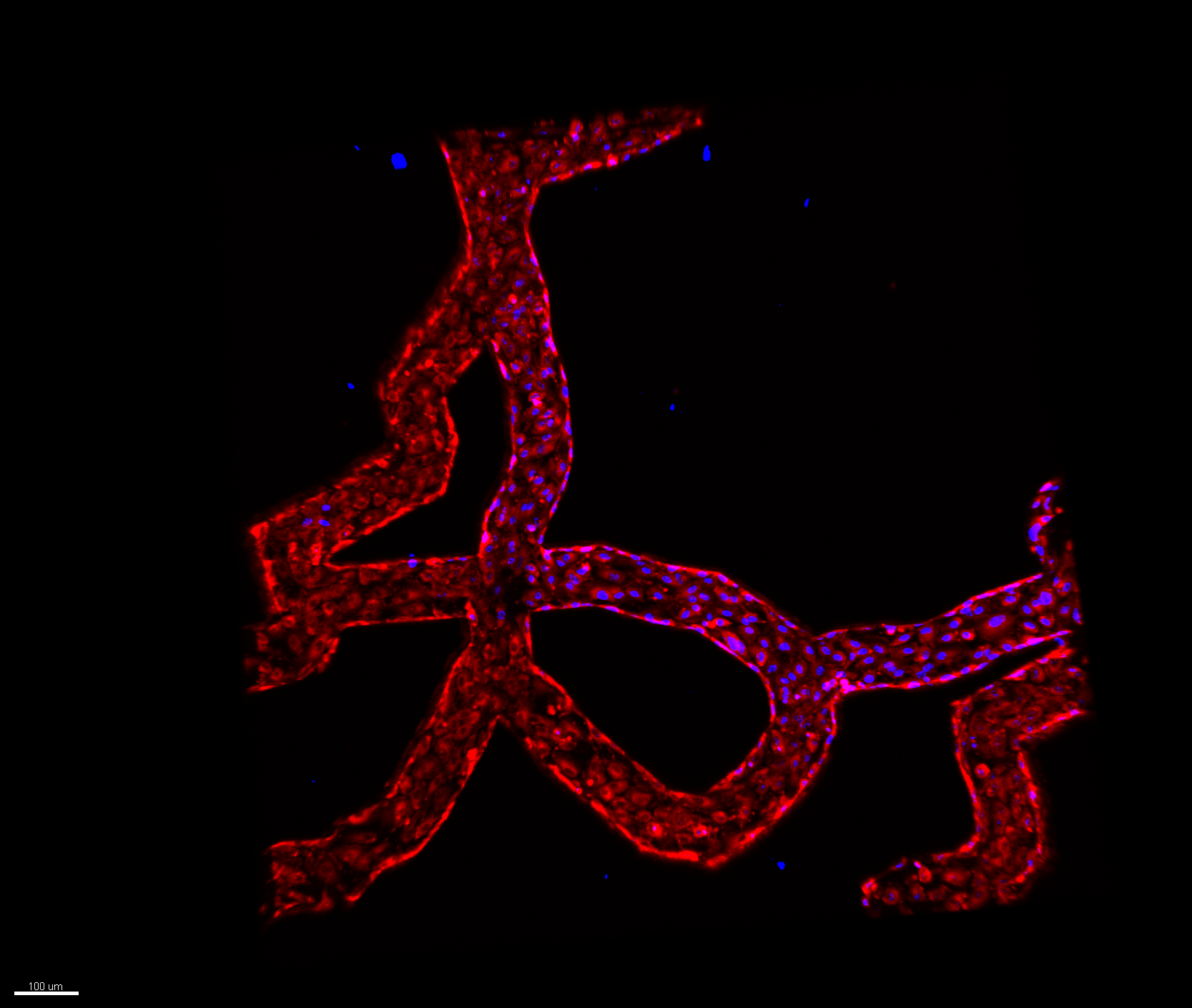
(b)
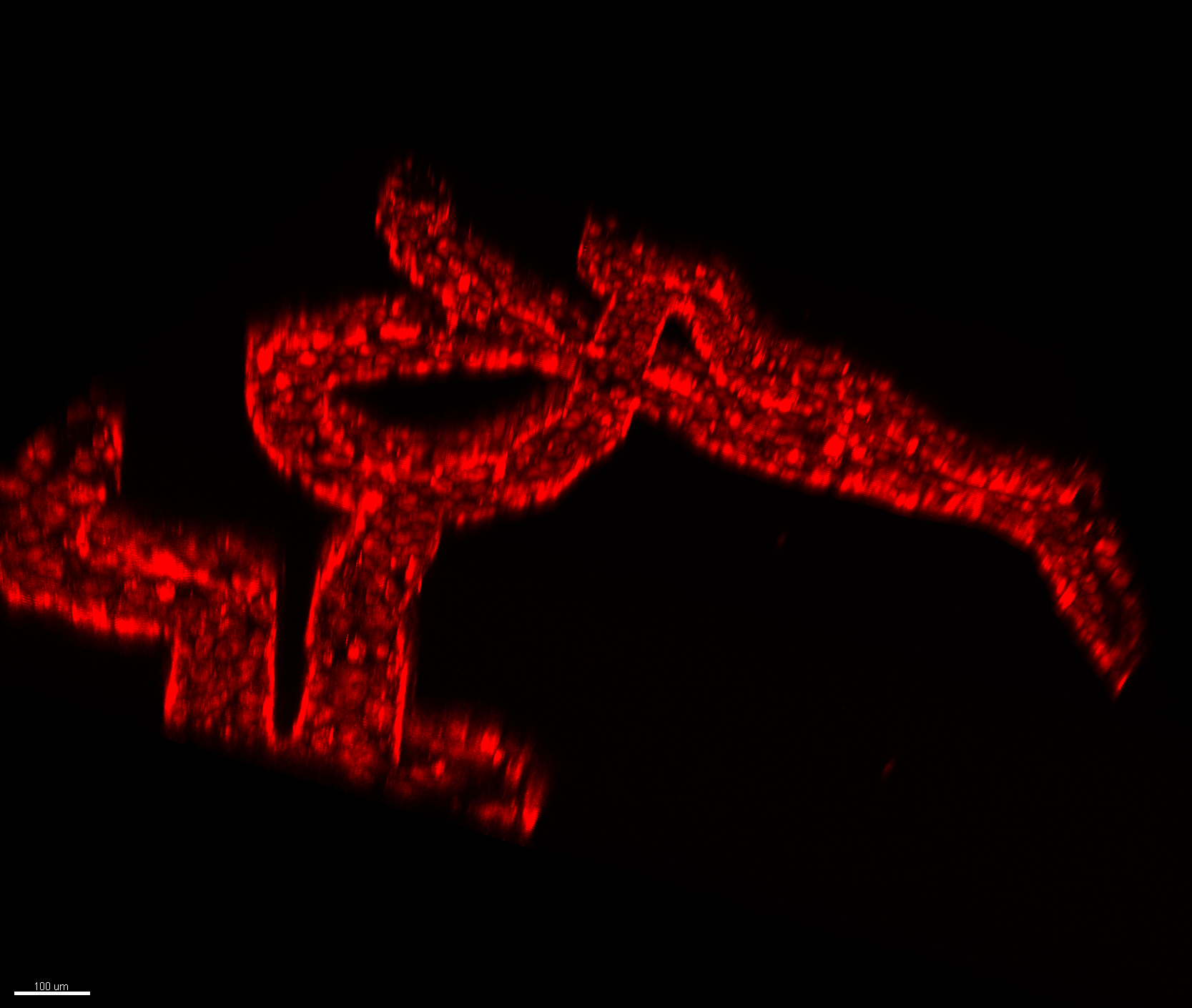
(c)
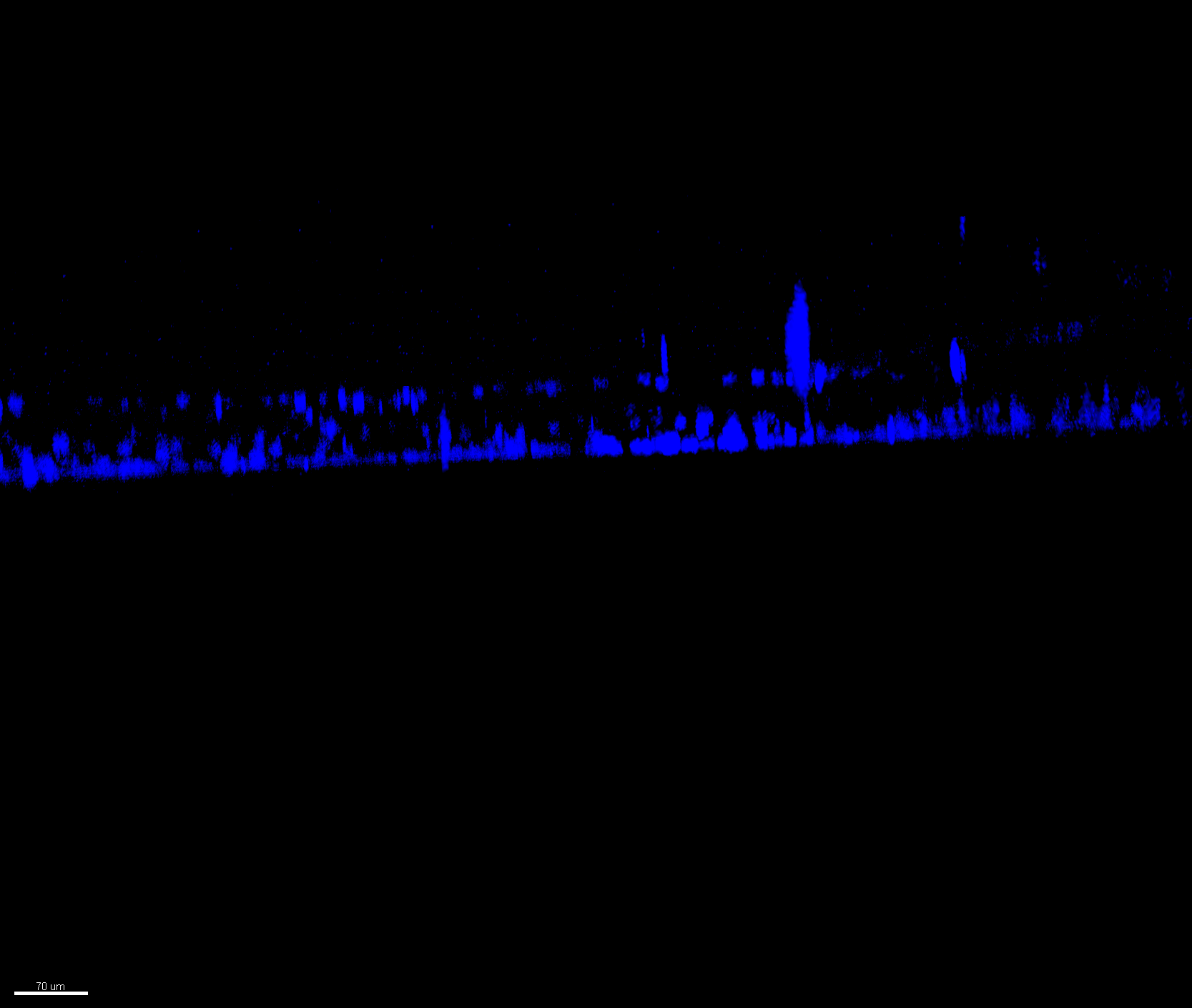
**

**(d)
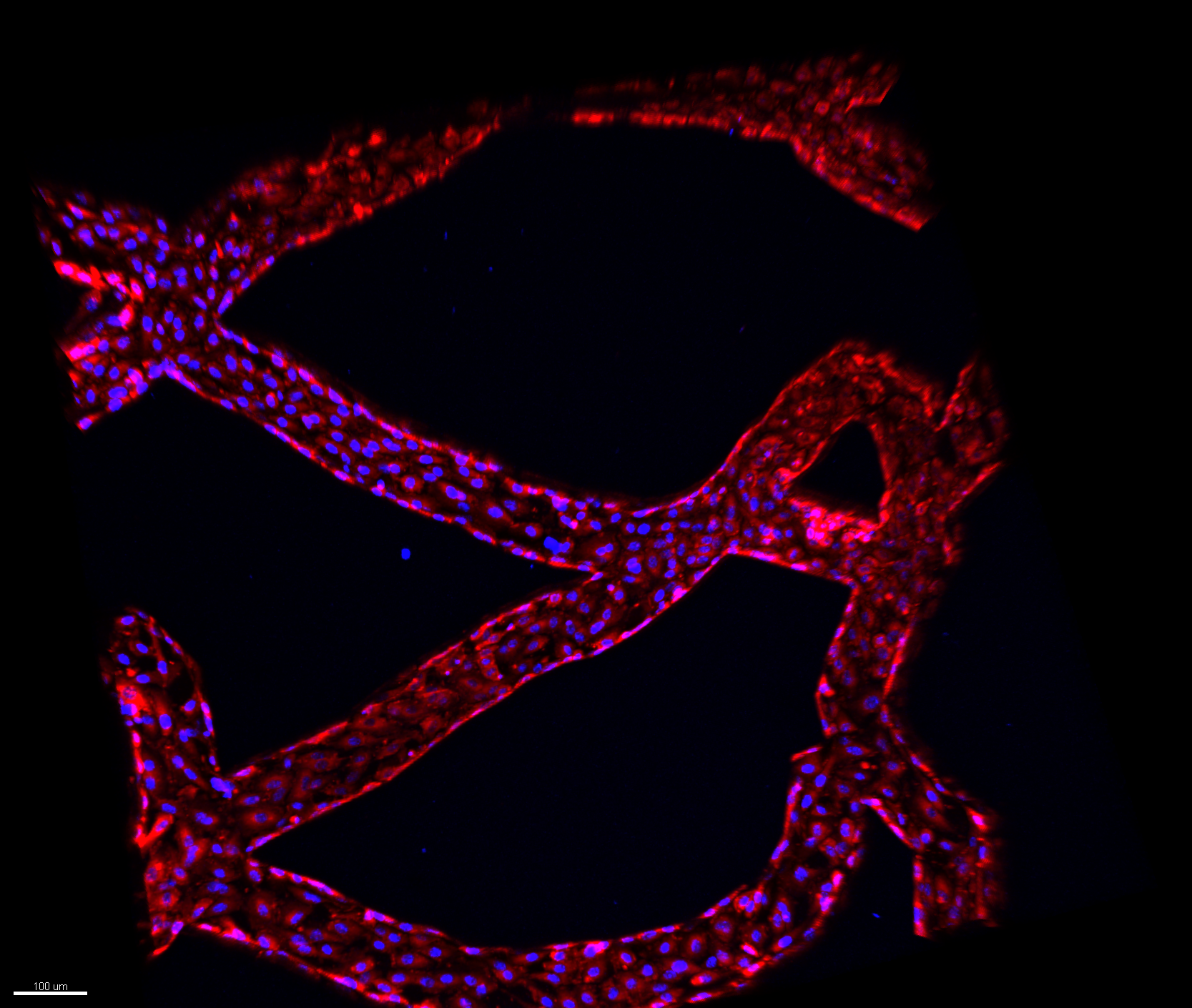
(e)
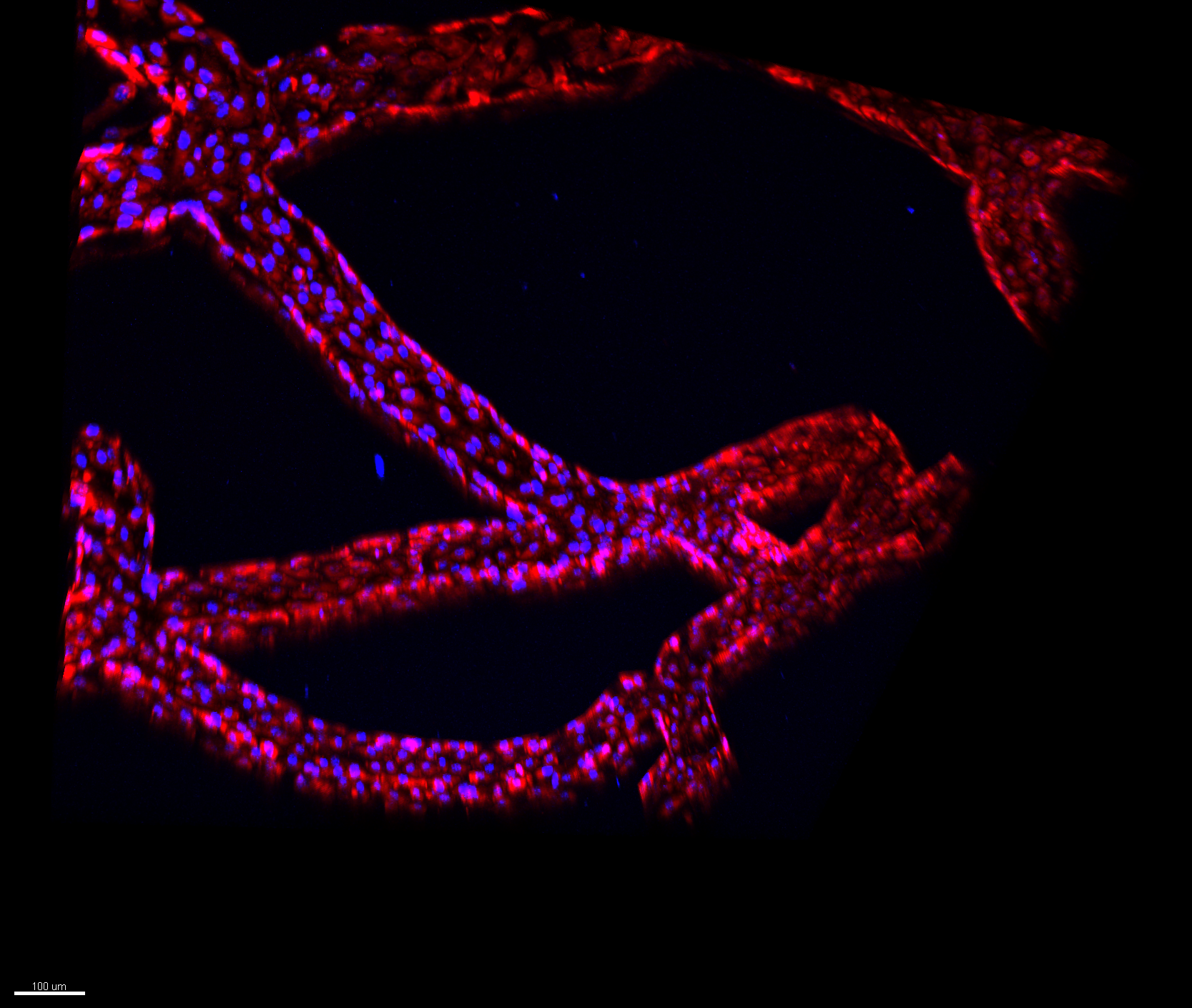
(f)
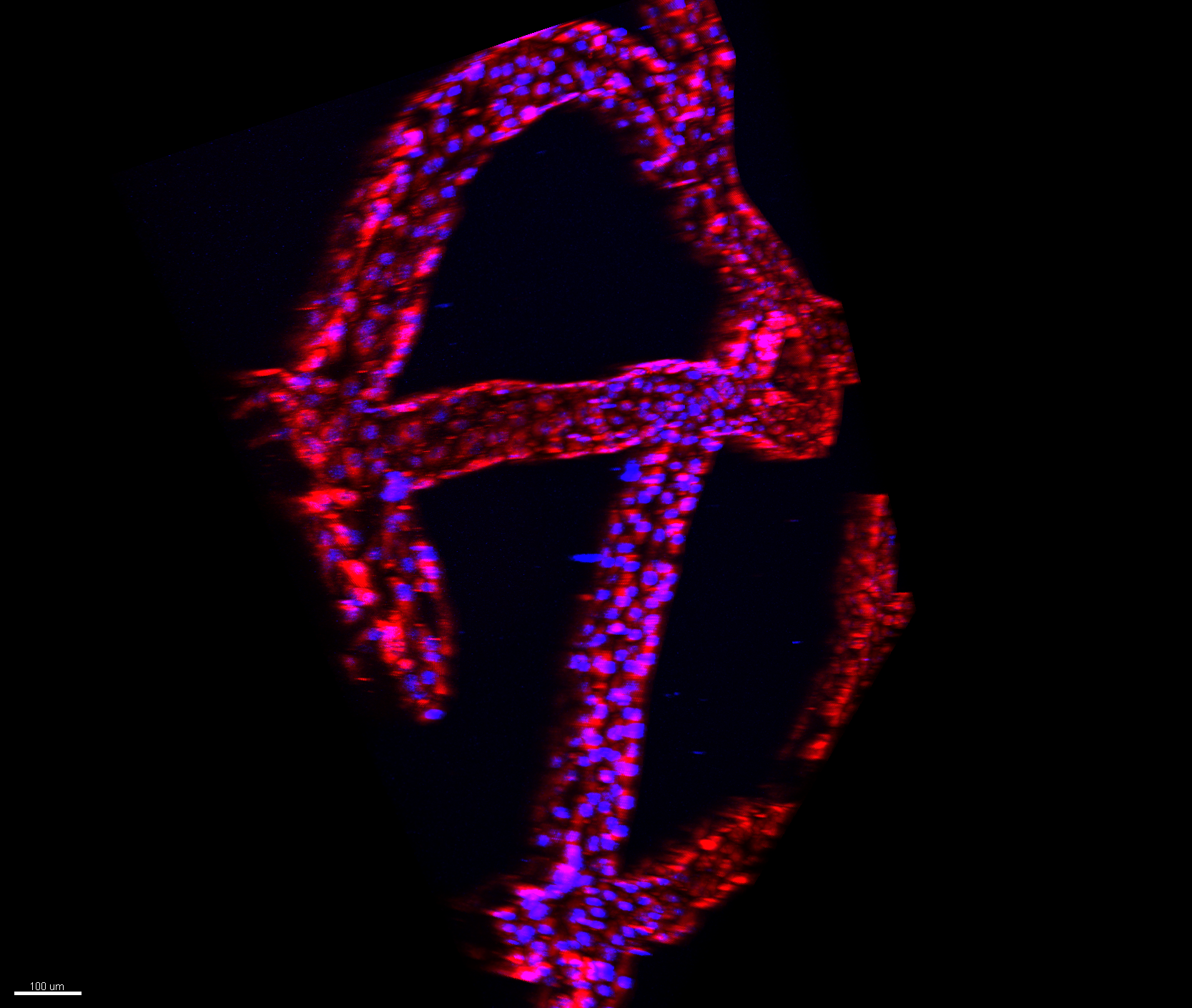
**

**Figure S2. Test MN’s stained with NucBlue (Blue) and primary and secondary antibodies for ZO-1 (Red) (a-f) different angles rendered to better view dimensionality of cell growth within chambers. Imaged using the multiarea time lapse feature on the Olympus Fluoview 1000 Spectral Confocal, stitched together using Fluoview software and stacked to create 3D rendering using Imaris imaging software.**

**Figure S3. Supernatant to pellet ratios of phosphorous content for stock nanocrystalline constructs (not subjected to flow).**
